# Multimaterial decomposition algorithm for quantification of fat in hepatocellular carcinoma using rapid kilovoltage-switching dual-energy CT: A comparison with chemical-shift MR imaging

**DOI:** 10.1097/MD.0000000000026109

**Published:** 2021-05-21

**Authors:** Takashi Ota, Masatoshi Hori, Kosuke Sasaki, Hiromitsu Onishi, Atsushi Nakamoto, Mitsuaki Tatsumi, Hideyuki Fukui, Kazuya Ogawa, Noriyuki Tomiyama

**Affiliations:** a Department of Diagnostic and Interventional Radiology, Osaka University Graduate School of Medicine; b CT Research Group, GE Healthcare, Japan.

**Keywords:** chemical shift imaging, hepatocellular carcinoma, liver, magnetic resonance imaging, tomography, X-ray computed

## Abstract

Understanding intratumoral fat in hepatocellular carcinoma (HCC) is clinically important to elucidate prognosis. We sought to quantify HCC and liver fat with a multimaterial decomposition (MMD) algorithm with rapid kilovoltage-switching dual-energy computed tomography (DECT) relative to chemical-shift magnetic resonance imaging (CSI).

In this retrospective study, 40 consecutive patients with HCC underwent non-contrast-enhanced (non-CE) and four-phases contrast-enhanced (four-CE) DECT (80 and 140 kVp) and abdominal MR imaging (including CSI) between April 2011 and December 2012. Fat volume fraction (FVF_DECT_) maps were generated by MMD algorithm to quantify HCC and liver fat. Fat fraction measured by CSI (FF_CSI_) was determined for HCC and liver on dual-echo sequence using 1.5- or 3-Tesla MR systems. The correlation between FVF_DECT_ and FF_CSI_ was evaluated using Pearson correlation test, while non-CE FVF_DECT_ and four-CE FVF_DECT_ were compared by one-way ANOVA and Bland–Altman analysis.

Forty patients (mean age, 70.1 years ± 7.8; 25 males) were evaluated. FVF_DECT_ and FF_CSI_ exhibited weak to moderate correlations for HCC in non-CE and four-CE except in equilibrium phase (*r* = 0.42, 0.44, 0.35, and 0.33; all *P* < .05), and very strong correlations for liver in all phases (*r* = 0.86, 0.83, 0.85, 0.87, and 0.84; all *P* < .05). Those correlation coefficients were significantly higher for liver for each phase (all *P* < .05). FVF_DECT_ did not differ significantly across scan phases regarding HCC or liver (*P* = .076 and 0.56). Bland–Altman analysis showed fixed bias in all phases between non- and four-CE FVF_DECT_ in HCC and liver.

As compared with liver, correlations between FVF measured by DECT-based MMD and FF measured by CSI were weak in HCC in all phases. FVF is reproducible across all scan phases in HCC and liver. The MMD algorithm requires modification for HCC fat quantification given the heterogeneous components of HCC.

## 1 Introduction

Intratumoral fat in hepatocellular carcinoma (HCC) may serve as an imaging biomarker supporting a more favorable prognosis according to prior research.^[1,2]^ Previously, fat-containing HCC was associated with less tumor-progression prevalence, less distant metastasis, and a longer time to tumor progression.^[1]^ Moreover, intratumoral fat detection in HCC may suggest a lower risk for microvascular invasion.^[2]^

Conventional single-energy computed tomography (CT) has been widely used to detect the fat component of the tissue in the clinical practice. The measurement of CT attenuation (expressed in Hounsfield units; HU) by drawing regions-of-interest (ROIs) is easy and convenient; however, this method is semiquantitative.^[3]^ Moreover, the fat component cannot be calculated in contrast-enhanced CT because the presence of contrast media alters the fat attenuation.^[4–5]^

Dual-energy computed tomography (DECT) technology facilitates the generation of material-specific images^[6–8]^ that display the distribution and concentration of a specific material (e.g., iodine, calcium, fat, uric acid) within the tissues.^[6]^ Consequently, these images provide visual and quantitative information about tissue composition.

A multimaterial decomposition (MMD) algorithm has been recently described to quantify liver fat for single-source rapid kilovoltage-switching DECT.^[9]^ The fat quantification is performed through dual-material decomposition using fat and healthy liver tissue as the material pair, and actual fat quantification is conducted with a convex-constrained least-squares problem.^[9]^ Moreover, the MMD algorithm can quantify the fat component in both non-contrast-enhanced (non-CE) and contrast-enhanced CT images.^[10]^ During contrast-enhanced CT imaging, virtual unenhancement (VUE) is preprocessing step of the liver fat quantification algorithm, which then proceeds to quantify the concentration of fat.^[9]^ A recent study by Hyodo et al showed that the MMD algorithm for liver fat quantification was accurate and reproducible across scan phases.^[11]^ Therefore, we hypothesized that MMD algorithm for HCC fat quantification is accurate as well as liver parenchyma. However, to our knowledge, intratumoral fat quantification in HCC has not been evaluated by the MMD algorithm so far.

This retrospective study sought to quantify intratumoral fat in HCC and fat in the liver with the MMD algorithm using rapid kilovoltage-switching DECT in comparison with chemical-shift magnetic resonance (MR) imaging (CSI) as the reference standard.

## 2 Materials and methods

### 2.1 Patient population

This retrospective study was approved by the institutional review board of Osaka University Hospital, which waived the requirement for informed consent. Between April 2011 and December 2012, 60 consecutive patients who underwent abdominal DECT (non-CE and four-phases contrast-enhanced CT [four-CE]) and abdominal MR imaging (including CSI) at Osaka University Hospital because of suspected HCC were enrolled. Some patients were subsequently excluded as follows:

1. technical issue (n = 1);
2. size of less than 1 cm (n = 2);
3. presence of diffuse-type HCC (n = 1);
4. no sign of HCC (n = 2);
5. disease other than HCC (i.e., combined-type HCC [n = 1], intrahepatic cholangiocellular carcinoma [n = 3], angiomyolipoma [n = 2]); and
6. interval of 70 days or more between DECT and MR examination (n = 1).

**Figure 1:**
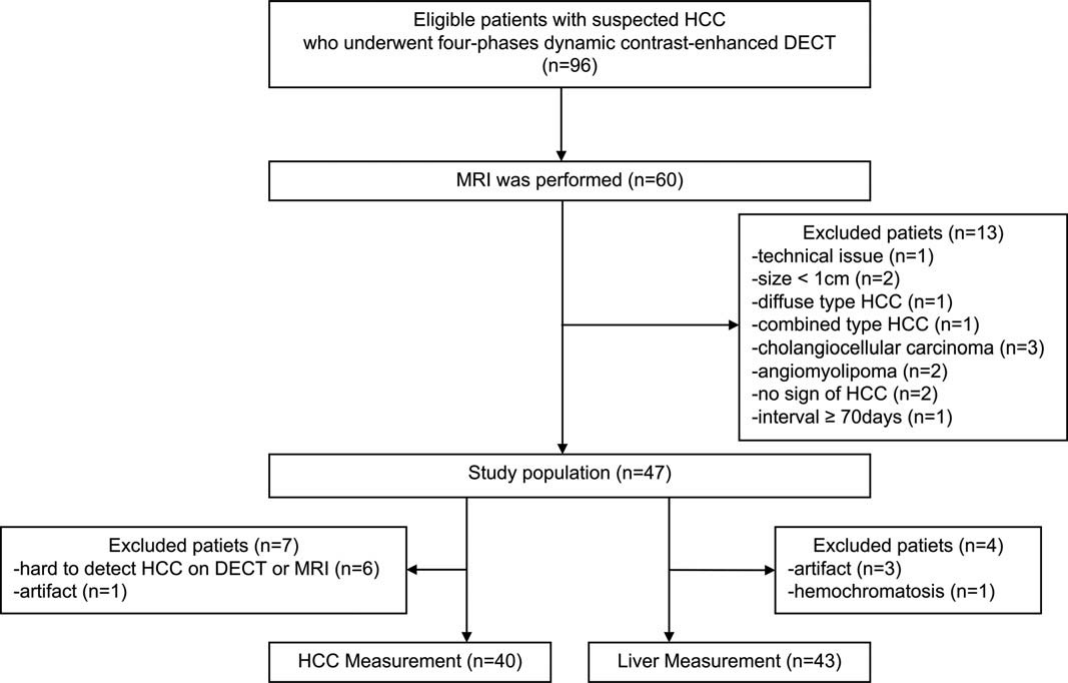
Flowchart of patient enrollment.

The final study population included 47 patients (mean ± standard deviation [SD] age: 70.1 ± 7.8 years; range: 53–87 years)—specifically, 28 men (mean ± SD age: 70.7 ± 7.1 years; range: 53–82 years) and 19 women (mean ± SD age: 70.9 ± 8.6 years; range: 55–87 years). One radiologist (board-certified radiologist with 8 years of experience in abdominal imaging) measured the maximum diameter and recorded the presence of each liver tumor, then assigned scores on CT images according to the Liver Imaging Reporting and Data System (LI-RADS) version 2018.^[12]^ We diagnosed liver tumors as HCC for LR-5 (definitely HCC) lesions. Of 47 patients, we measured 40 HCCs in 40 patients (if multiple tumors were seen, we selected the largest tumor for consideration) and 43 livers. Excluded patients are listed in Figure 1. Of 40 patients, 26 patients underwent surgical resection, 10 patients underwent transcatheter arterial chemoembolization (TACE), 2 patients underwent chemotherapy (sorafenib), and 2 patients underwent other therapy.

### 2.2 Dual-energy CT imaging

All DECT images were acquired using a 64-channel multi-detector CT scanner (Discovery CT750HD; GE Healthcare, Waukesha, WI). Scan parameters are listed in Table 1. Following non-CE data acquisition, patients were administrated contrast material containing 350 mg/mL iodine (Iomeron 350; Eisai, Tokyo, Japan). The amount and injection duration of the contrast material and scan timing are presented in Table 1. The MMD algorithm is a commercially unavailable postprocessing software (Liver Fat Quantification; GE Healthcare). Liver fat quantification algorithm was developed by using the MMD algorithm with fat, liver tissue, and blood in the material basis.^[9]^ In the contrast-enhanced DECT data, a virtual unenhancement image was firstly applied before running liver fat quantification algorithm. In this step, the iodine contrast material has been removed and replaced by the same volume blood. The final output is the fat volume fraction (FVF) map.

**Table 1:** Scan parameters and scan protocol of rapid kilovoltage-switching dual-energy CT.

CT scanner	Discovery CT 750HD
Scan parameters of DECT	
Energy level	80/140 kVp fast-switching, 630 mA
Rotation time	0.5 second
Helical pitch	1.375: 1
Image thickness	5.0 mm
Beam width	40 mm (detector coverage)
CT dose index	12.72 mGy
Contrast material and scan timing	
Amount of contrast material	1.715 mL/kg containing 350 mg/ml iodine
Injection duration of contrast material	26 seconds (in antecubital vein through 20-gauge plastic cannula)
Scan phase	Scan timing
Early arterial phase	8 seconds after the attenuation in abdominal aorta reached 100 HU
Late arterial phase	10 seconds after early arterial phase
Portal venous phase	30 seconds after late arterial phase
Equilibrium phase	120 seconds after portal venous phase

### 2.3 Chemical-shift MR imaging

Of 47 patients, 28 patients were examined using 3.0-Tesla (T) MR system (Signa EXCITE HDxt; GE Healthcare), 12 patients were examined using 3.0-T MR system (Achieva; Philips Medical Systems, Best, the Netherlands), and 7 patients were examined using 1.5-T MR system (Signa EXCITE HD; GE Healthcare). The scan parameters are summarized in Table 2.

**Table 2:** Scan parameters of chemical-shift imaging by using 3 MR systems.

MR system	Signa EXCITE HDxt	Achieva	Signa EXCITE HD
Subject number	28	12	7
TR/TE (in-phase)	5.7/2.7 msec	4/2.5 msec	200/4.5 msec
TR/TE (opposed-phase)	5.7/1.3 msec	4/1.2 msec	200/2.3 msec
Slice thickness	4 mm (Zip 2)	5 mm	5 mm
Slice spacing	N/A	0 mm	2 mm
Flip angle	12°	10°	90°
FOV	34 × 34 cm	35 × 35 cm	34 × 34 cm
Matrix	320 × 192	160 × 192	320 × 192
Bandwidth	651 kHz	943 kHz	325 kHz

### 2.4 Image analysis

**Figure 2:**
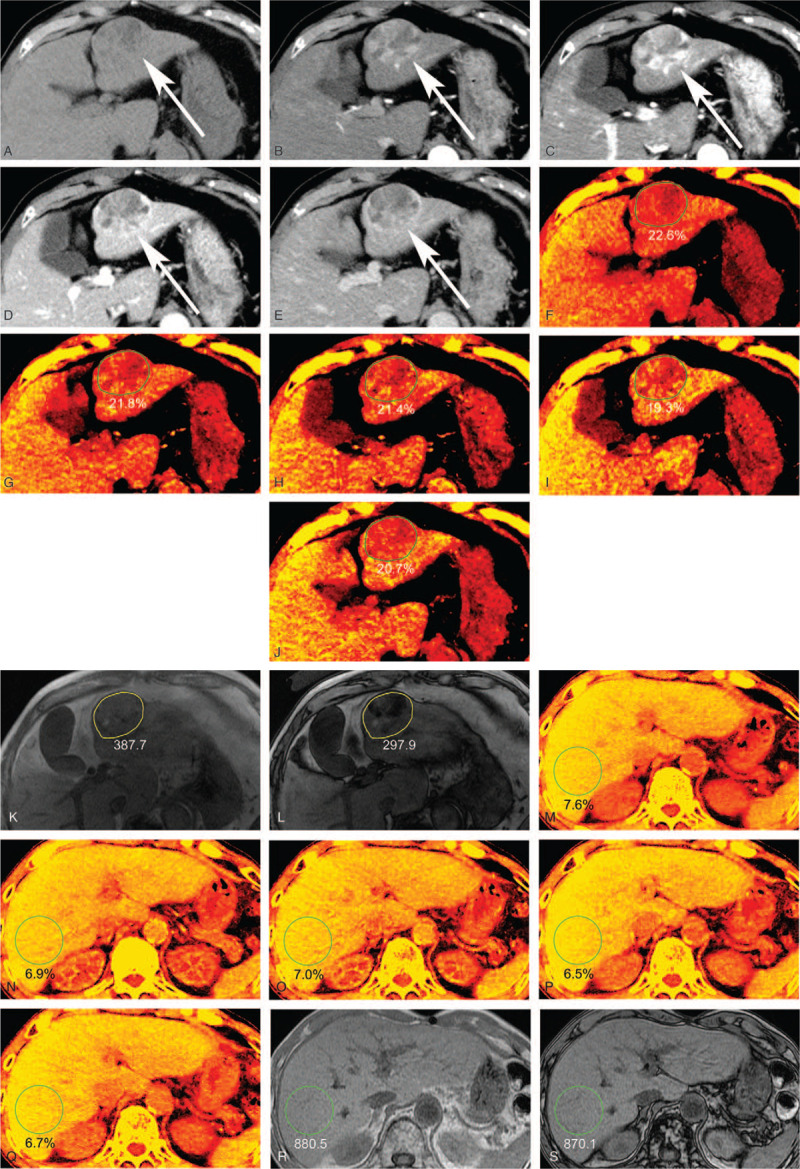
A 79-year-old male with HCC with fat component. Non- and four-CE CT (A–E) and FVF maps by using the MMD algorithm in each phase (F–J) and in-phase and opposed-phase images (K, L). (A) Non-CE CT shows a 5-cm-sized hepatic mass with a hypodense area in segment 3 (arrow). (B, C) On AP1 and AP2, the tumor shows nonrim arterial hyperenhancement (arrow). (D, E) On PVP and EP, the tumor shows nonperipheral “washout” (arrow). Enhancing capsule appearance is also seen in EP. The tumor is categorized as LR-5 (definitely HCC) by using the LI-RADS version 2018. (F) In the FVF map, FVF (%) of the tumor shows (F) 22.6% in non-CE, (G) 21.8% in AP1, (H) 21.4% in AP2, (I) 19.3% in PVP, and (J) 20.7% in EP by placing freehand ROIs (green circle). (K) Signal intensities of the tumor are 387.7 on in-phase image and (L) 297.9 on opposed-phase image by placing freehand ROIs (yellow circle). FF_CSI_ yields 11.6%. Surgical pathological findings confirmed “moderately differentiated HCC.” A 74-year-old male with HCC. FVF maps by using the MMD algorithm in each phase (M–Q) and in-phase and opposed-phase images (R, S). Circle ROI was placed on the right robe homogeneous area. In the FVF map, FVF (%) of the liver parenchyma shows (M) 7.6% in non-CE, (N) 6.9% in AP1, (O) 7.0% in AP2, (P) 6.5% in PVP, and (Q) 6.7% in EP by placing freehand ROIs (green circle). (R) Signal intensities of the liver are 880.5 on in-phase image and (S) 870.1 on opposed-phase image by placing circle ROIs (yellow circle). FF_CSI_ yields 0.6%. AP1 = early arterial phase, AP2 = late arterial phase, EP = equilibrium phase, FF_CSI_ = fat fraction calculated from chemical shift imaging, four-CE = four-phases contrast-enhanced, FVF = fat volume fraction, HCC = hepatocellular carcinoma, MMD = multimaterial decomposition, non-CE = non-contrast-enhanced, PVP = portal venous phase.

All DECT FVF maps and CSI were anonymized and transferred to an image viewer (EV Insite S; PSP, Tokyo, Japan). The aforementioned radiologist placed HCC ROIs on FVF maps or CSI. Freehand ROIs designated HCC lesions on the FVF map (non-CE data), referring to the four-CE CT images, then were copied to the VUE FVF maps at the same level. Freehand ROIs were drawn along the tumor borders to cover the entire tumor area on slices showing the maximum tumor size. Particular care was taken to exclude inhomogeneous areas (e.g., necrosis, bleeding). By placing ROIs, fat component inside can be directly measured in percentage volume (fat volume fraction measured by DECT-based MMD algorithm (FVF_DECT_) (Fig. 2).

Freehand ROIs were also placed on HCC lesions on opposed-phase (OP) images and copied to in-phase (IP) images at the same level. Care was taken to place ROIs on FVF maps and CSI at the same or similar levels wherever possible. Signal intensities were obtained by drawing ROIs both on IP and OP images. Fat fraction measured by CSI (FF_CSI_) was calculated using the following equation (Fig. 2)^[13]^:

$$FF_{CSI}=[(IP-OP)/(2\times IP)]\times 100^{\left[13\right]}$$

The radiologist also placed circular ROIs of the liver on FVF maps (non-CE and four-CE) or CSI. Care was taken to exclude large vessels, liver edges, and artifacts (Fig. 2). By placing ROIs on liver, we calculated the FVF_DECT_ and FF_CSI_ in the liver among 43 patients.

### 2.5 Statistical analysis

Correlation between FVF_DECT_ and FF_CSI_ was evaluated by using Pearson correlation coefficient. Comparisons between 2 correlations (HCC vs liver) in each scan phase were examined by transforming the correlation coefficient into Z-scores. Non-CE FVF_DECT_ and four-CE FVF_DECT_ data were compared to determine the reproducibility of MMD by Bland–Altman analysis and one-way analysis of variance (ANOVA), and *P* values of less than .05 were considered as statistically significant. All statistical analyses were performed using SPSS version 24 (IBM, Armonk, NY).

## 3 Results

### 3.1 Histologic, imaging findings, and tumor staging

Twenty-six patients’ liver tumors were surgically resected and all tumors were histologically diagnosed as HCC. Of these 26 tumors, 6 were well-differentiated, 16 were moderately-differentiated, and 4 were poorly-differentiated HCC.

During dynamic contrast-enhanced CT imaging, all tumors (n = 40) showed arterial-phase hyperenhancement (APHE), 37 tumors showed nonperipheral “washout,” and 28 tumors showed enhancing “capsules.” the average tumor observation size was 47.6 ± 31.6 mm (range: 15–198 mm). All tumors were categorized as LR-5 (i.e., definitely HCC) by using the LI-RADS version 2018. Of 40 patients, 3 were stage IA, 2 were stage IB, 20 were stage II, 7 were stage IIIA, 5 were stage IIIB, 1 was stage IVA and 2 patients were categorized as stage IVB according to American Joint Committee on Cancer (AJCC) 8th edition staging system.

### 3.2 Comparison of FVF_DECT_ and FF_CSI_ in HCC

**Figure 3:**
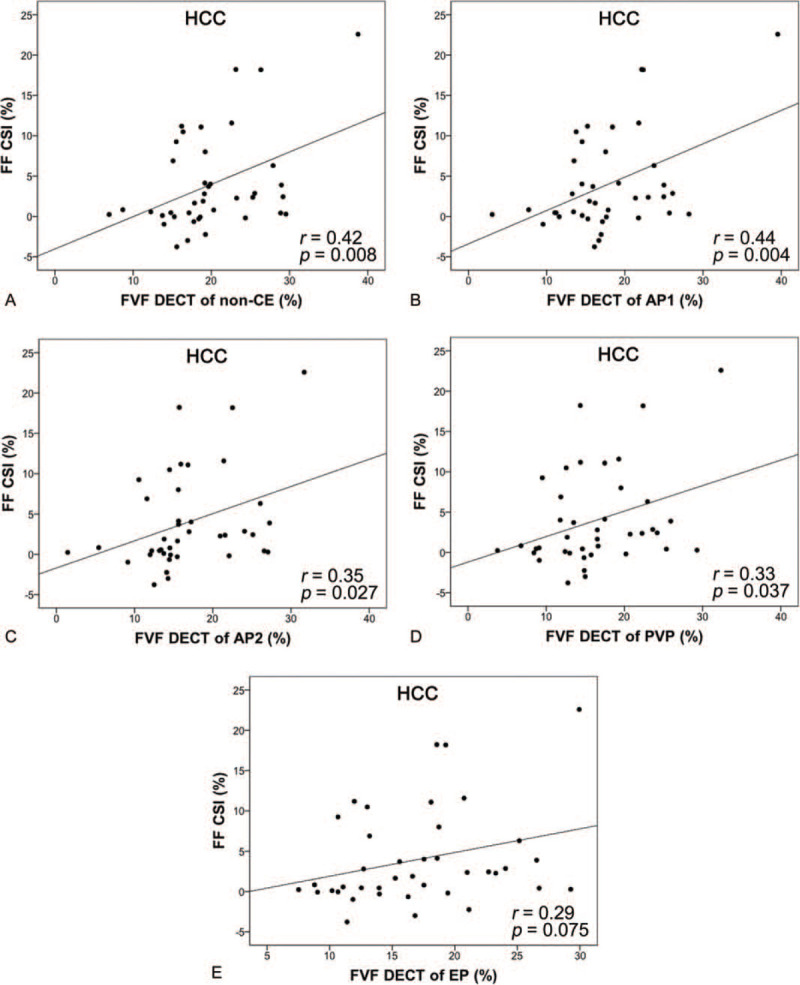
Scatter plots (HCC) of FVF measured by MMD algorithm (FVF_DECT_) (horizontal line) vs FF measured by CSI (FF_CSI_) (vertical line). The line represents linear regression. (A) Non-CE, (B) early-arterial phase, (C) late-arterial phase, (D) portal-venous phase, and (E) equilibrium-phase images can be seen. The correlations between FVF_DECT_ and FF_CSI_ were weak to moderate in each phase except the equilibrium phase, where a significant correlation was not observed between FVF_DECT_ and FF_CSI_. CSI = chemical-shift magnetic resonance imaging, FF = fat fraction, FVF = fat volume fraction, MMD = multimaterial decomposition, non-CE = non-contrast-enhanced.

FVF_DECT_ and FF_CSI_ values of HCCs were compared by Pearson correlation coefficient in all phases and were as follows: non-CE, *r* = 0.42 (*P* = .008); early arterial phase, *r* = 0.44 (*P* = .004); late arterial phase, *r* = 0.35 (*P* = .027); portal venous phase, *r* = 0.33 (*P* = .037); and equilibrium phase, *r* = 0.29 (*P* = .075). Non-CE as well as early-arterial, late-arterial, and portal-venous phase imaging showed statistically significant weak to moderate correlations between FVF_DECT_ and FF_CSI_. The equilibrium phase showed no correlation between FVF_DECT_ and FF_CSI_ (Fig. 3).

### 3.3 Comparison of FVF_DECT_ and FF_CSI_ in liver parenchyma

**Figure 4:**
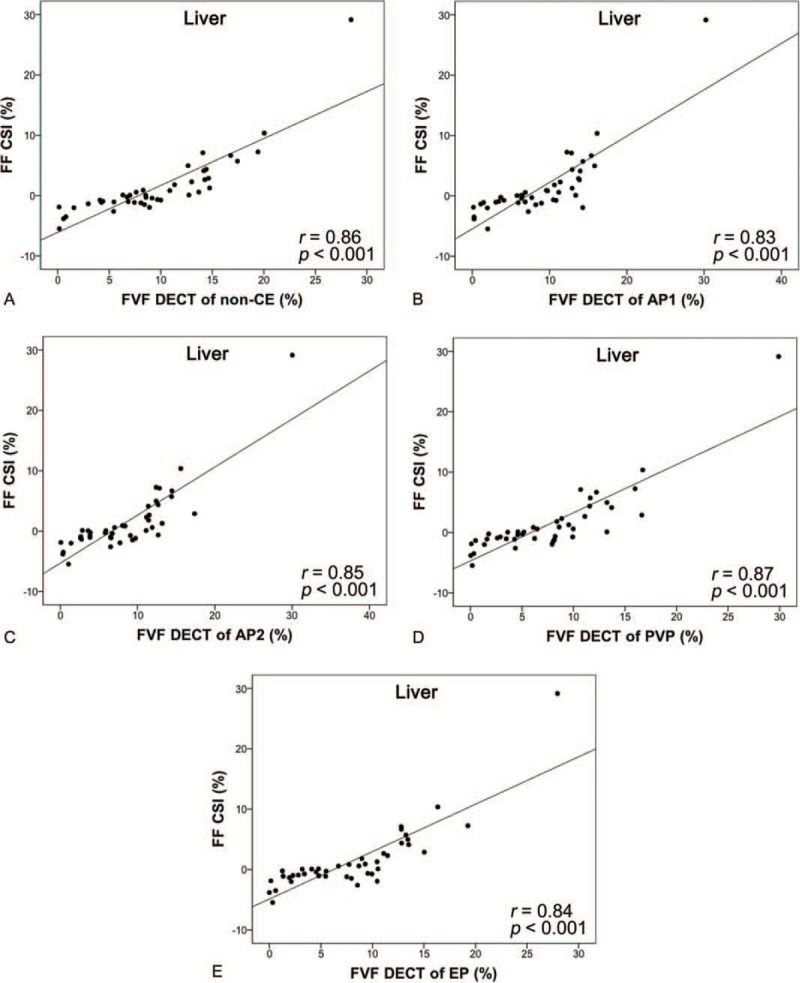
Scatter plots (liver parenchyma) of FVF measured by the MMD algorithm (FVF_DECT_) (horizontal line) vs FF measured by CSI (FF_CSI_) (vertical line). The line represents linear regression. (A) Non-CE, (B) early-arterial phase, (C) late-arterial phase, (D) portal-venous phase, and (E) equilibrium-phase images can be seen. The correlations between FVF_DECT_ and FF_CSI_ were very strong in all phases. CSI = chemical-shift magnetic resonance imaging, FF = fat fraction, FVF = fat volume fraction, MMD = multimaterial decomposition, non-CE = non-contrast-enhanced.

The FVF_DECT_ and FF_CSI_ values of liver parenchyma were also compared by Pearson correlation coefficient in all phases and were as follows: non-CE, *r* = 0.86 (*P* < .001); early arterial phase, *r* = 0.83 (*P* < .001); late arterial phase, *r* = 0.85 (*P* < .001); portal venous phase, *r* = 0.87 (*P* < .001); and equilibrium phase, *r* = 0.84 (*P* < .001). Non-CE imaging and that in all 4 phases showed very strong correlations between FVF_DECT_ and FF_CSI_ (Fig. 4).

### 3.4 Pearson correlation coefficient comparisons between HCC and liver parenchyma

Pearson correlation coefficient of liver parenchyma was significantly higher than that of HCC in all phases as follows: non-CE, Z-score = 3.65 (*P* < .001); early arterial phase, Z-score = 3.12 (*P* = .001); late arterial phase, Z-score = 3.83 (*P* < .001); portal venous phase, Z-score = 4.25 (*P* < .001); and equilibrium phase, Z-score = 4.12 (*P* < .001) (Table 3).

**Table 3:** Comparison of Pearson correlation coefficient between HCC and liver parenchyma on non-CE and four-CE phases.

	HCC	Liver parenchyma	Z-score	*P* value
Non-contrast enhanced	0.42	0.86	3.65	<.001
Early arterial phase	0.44	0.83	3.12	.001
Late arterial phase	0.35	0.85	3.83	<.001
Portal venous phase	0.33	0.87	4.25	<.001
Equilibrium phase	0.29	0.84	4.12	<.001

### 3.5 FVF difference between non-CE and VUE images in each phase in MMD

**Figure 5:**
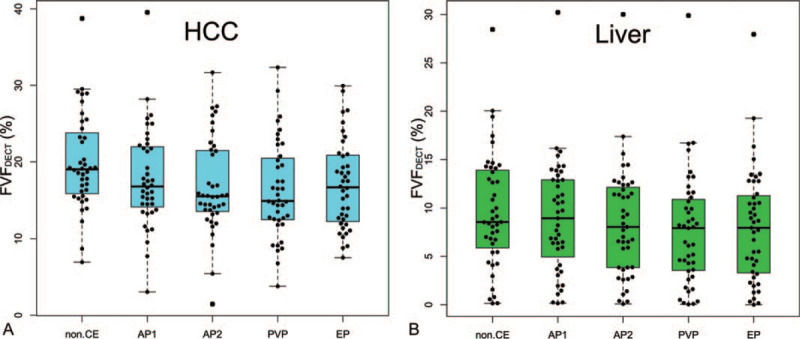
Box plots of FVF (vertical line) measured with DECT for (A) HCC and (B) liver parenchyma of different scan phases (horizontal line). The center line of the box plot is the mean value, the box represents the 95% CI (confidence interval), and whiskers represent the range of the values. FVF did not significantly differ by one-way ANOVA among each phase for both HCC and liver parenchyma (*P* = .076 and 0.56). AP1 = early arterial phase, AP2 = late arterial phase, DECT = dual-energy CT, EP = equilibrium phase, FVF = fat volume fraction, non-CE = non-contrast-enhanced, PVP = portal venous phase.

Box plots of FVF_DECT_ for all scan phases of HCC and liver parenchyma are shown in Figure 5. FVF_DECT_ did not significantly differ by one-way ANOVA among each phase for either HCC or liver parenchyma (*P* = .076 and .56). The mean FVF_DECT_ values of HCC and liver parenchyma are shown in Table 4.

**Table 4:** Mean fat volume fraction values measured by DECT (FVF_DECT_) of HCC and liver parenchyma.

	HCC		Liver parenchyma	
Phase	FVF_DECT_ (%)	FF_CSI_ (%)	FVF_DECT_ (%)	FF_CSI_ (%)
	Mean ± SD (95% CI)	Mean ± SD (95% CI)	Mean ± SD (95% CI)	Mean ± SD (95% CI)
Non-CE	20.0 ± 6.2 (18.0–22.0)		9.6 ± 5.9 (7.8–11.5)	
AP1	17.8 ± 6.4 (15.8–19.9)		8.9 ± 5.8 (7.1–10.7)	
AP2	16.8 ± 6.2 (14.8–18.8)		8.5 ± 5.7 (6.7–10.2)	
PVP	16.3 ± 6.3 (14.3–18.3)		7.7 ± 5.9 (5.9–9.5)	
EP	17.0 ± 5.8 (15.2–18.9)		8.0 ± 5.8 (6.2–9.8)	
CSI		4.0 ± 6.0 (2.0–5.9)		1.4 ± 5.4 (–0.3 to –3.1)

**Figure 6:**
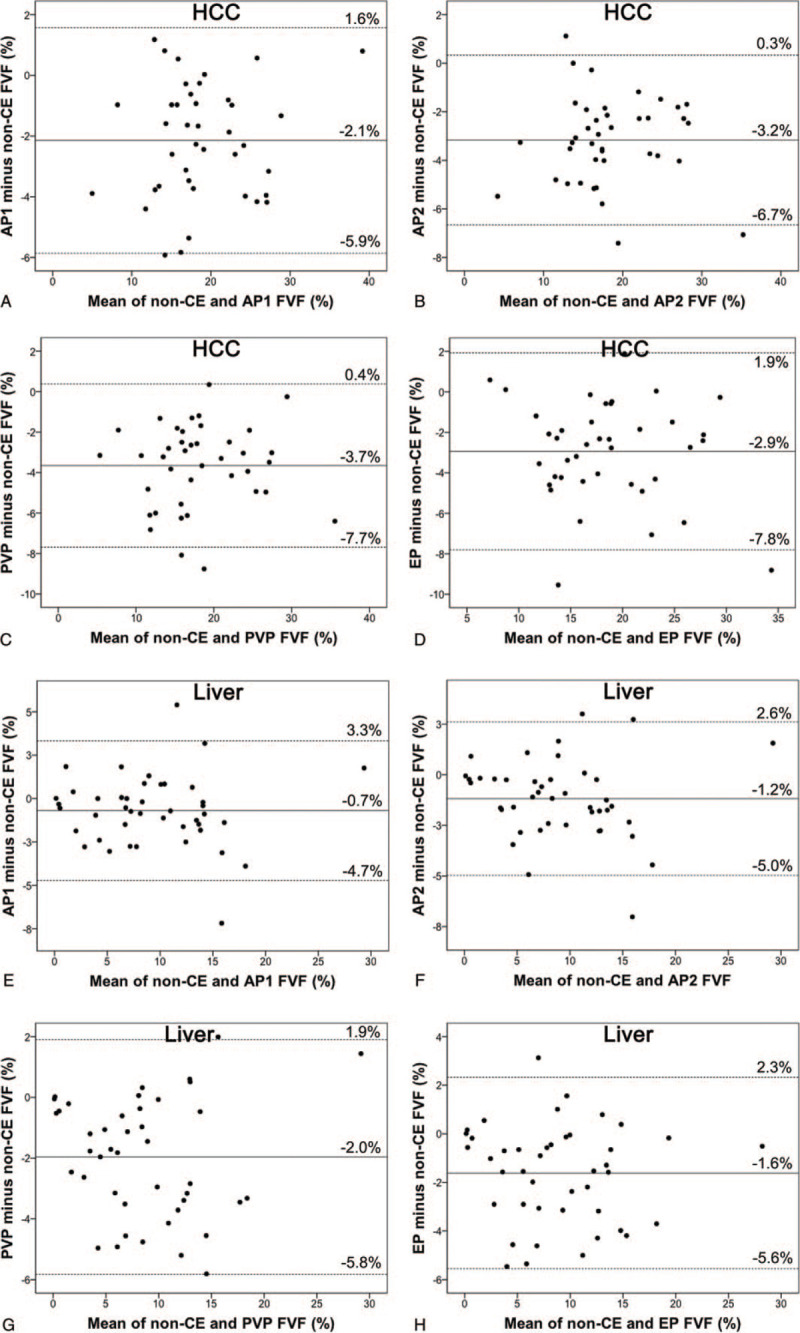
Bland–Altman plots of HCC (A–D) and liver parenchyma (E–H) of average FVF values calculated from non-CE and post-CE FVF maps (horizontal line) vs the difference between post- and non-CE FVF values (vertical line). The continuous line represents the mean absolute difference (bias) in FVF values between non-CE and post-CE images; dashed lines represent the upper and lower limits of agreement. Graphs (A–D) show limits of agreement in HCC between FVF assessed by non-CE and those assessed by (A) AP1, (B) AP2, (C) PVP, and (D) EP. As for HCC, fixed bias was seen in all phases. Graphs (E–H) show limits of agreement in liver parenchyma between FVF assessed by non-CE and those assessed by (E) AP1, (F) AP2, (G) PVP, (H) EP. As for liver parenchyma, fixed bias was seen in all phases; however, the mean differences were smaller than HCC in all phases. AP1 = early arterial phase, AP2 = late arterial phase, EP = equilibrium phase, FVF = fat volume fraction, non-CE = non-contrast-enhanced, post-CE = post-contrast-enhanced, PVP = portal venous phase.

Bland–Altman analyses of FVF_DECT_ data and each phase of contrast-enhanced CT data were also evaluated. Fixed bias was seen in all 4 phases in FVF_DECT_ data between pre- and post-contrast-enhanced FVF_DECT_ data in HCC and liver parenchyma. As for HCC, mean differences between post-contrast and pre-contrast were as follows: early arterial phase, −2.1% (95% CI: −2.8% to −1.5%); late arterial phase, −3.2% (95% CI: −3.7% to −2.6%); portal venous phase, −3.7% (95% CI: −4.3% to −3.0%); and equilibrium phase, −2.9% (95% CI: −3.7% to −2.1%). The upper and lower limits of agreement were as follows: early arterial phase, 1.6% and −5.9%; late arterial phase, 0.3% and −6.7%; portal venous phase, 0.4% and −7.7%; and equilibrium phase, 1.9% and −7.8% (Fig. 6).

As for liver parenchyma, the mean differences between post-contrast and pre-contrast were as follows: early arterial phase, −0.7% (95% CI: −1.3% to −0.6%); late arterial phase, −1.2% (95% CI: −1.8% to −0.6%); portal venous phase, −2.0% (95% CI: −2.6% to −1.4%); and equilibrium phase, −1.6% (95% CI: −2.2% to −1.0%). The upper and lower limits of agreement were as follows: early arterial phase, 3.3% and −4.7%; late arterial phase, 2.6% and −5.0%; portal venous phase, 1.9% and −5.8%; and equilibrium phase, 2.3% and −5.6% (Fig. 6).

## 4 Discussion

In the clinical image interpretation, “fat measurement” plays a crucial role in accurately diagnosing and discerning malignancy of the tumor. In abdominal lesions, observation of the fat component during imaging sometimes leads to the correct diagnosis (e.g., see adrenal adenoma and myelolipoma, renal angiomyolipoma, and ovarian teratoma). Especially, fat-containing HCC shows a more favorable prognosis and reduced prevalence of microvascular invasion relative to nonfat-containing HCC.^[1–2]^ Hence, fat measurement is clinically important to making a radiological diagnosis and speculating the prognosis, especially for HCC. In the clinic, ultrasonography (US), CT, and MR imaging are useful modalities by which to quantify the fat component. In recent studies, researchers suggested the DECT-based MMD algorithm can quantify liver fat accurately.^[10–11]^ Therefore, we adapted this algorithm to HCC fat quantification. However, we demonstrated that the performance of the MMD algorithm is relatively poorer in HCC than in liver parenchyma.

A recent study by Hur et al showed that FVFs calculated by the MMD algorithm in post-contrast-enhanced (post-CE) DECT were strongly correlated with those of pathology as well as chemical-shift imaging for obtaining the proton-density fat fraction (PDFF) in 16 rabbits’ livers (*r* = 0.794 and 0.652).^[10]^ In our data, FVFs calculated by MMD analysis were very strongly correlated with FF calculated by dual-echo CSI (non-CE: *r* = 0.86; post-CE: *r* = 0.83–0.87) in 43 patients’ livers. In comparison, FVF had a higher correlation with MR imaging. This difference is partly because of subject number (16 vs 43), partly because of MR sequence (PDFF vs CSI), and partly because of the difference in species (rabbit vs human). Another recent study by Hyodo et al revealed that both FVFs calculated by MMD and MRI spectroscopy FF increased with rising histologic steatosis grade (trend test, *P* < .001 for each) in 37 patients’ livers.^[11]^ We did not conduct pathological evaluations; however, we demonstrated a very strong positive correlation between FVF calculated by DECT and FF calculated by CSI, so similar trends might be seen between the 2 techniques and pathological results.

On the other hand, FVF measured by DECT and FF measured by CSI had weak to moderate correlations in HCC (non-CE: *r* = 0.42; post-CE: *r* = 0.29–0.44). This relatively poor performance of the MMD algorithm is mainly due to its role as a “liver fat quantification algorithm.” In this algorithm, fat is measured by using fat, liver tissue, and blood on a material basis.^[9]^ Naturally, involving other material skews the FVF values measured by the MMD algorithm. Liver parenchymas almost purely consist of liver tissue, blood, and fat. On the other hand, HCC cells are polygonal, having granular and eosinophilic cytoplasm with nuclear pleomorphism and a high nuclear to cytoplasmic ratio. These tumor cells may secrete bile and contain fat, glycogen, Mallory–Denk bodies, hyaline globules, or fibrinogen.^[14]^ In this regard, these tumor cells are very different from normal liver cells. In HCC, the normal liver cells are replaced by multiplying tumor cells. Various components other than liver tissue, blood, and fat within HCC might skew the FVF values in the MMD algorithm. We estimate the weak correlation between DECT and CSI in HCC is mainly due to this consideration.

As for the FVF difference between non-CE and VUE images in each phase in the MMD algorithm, FVF did not significantly differ in HCC and liver parenchyma in all comparisons of scan phases by one-way ANOVA (*P* = .076 and .56). Bland–Altman plots revealed that fixed bias was present in all phases in both HCC and liver parenchyma. However, the mean bias was smaller in liver parenchyma than in HCC in each phase (early arterial phase: −0.69% vs −2.14%; late arterial phase: −1.18% vs −3.17%; portal venous phase: −1.96% vs −3.65%; and equilibrium phase: −1.61% vs −2.93%). These results suggest that the VUE step is performed more accurately in liver parenchyma than in HCC. This is further supported by the fact that, relative to liver parenchyma, the enhancement pattern of HCC is strong, variable, and heterogeneous. Hyodo et al demonstrated that contrast-enhanced FVF tended to present higher values than non-CE FVF in Bland–Altman plots.^[11]^ On the other hand, our data showed that contrast-enhanced FVF tended to achieve lower values than non-CE FVF. The VUE step in their study might be differently performed from our study; however, we cannot fully explain this discrepancy. Contrast-material removal by means of the VUE step is a key component during fat quantification with the MMD algorithm. We assume that the VUE step must be modified to account for different contrast-enhancement patterns among the phases, especially for HCC.

Our study had some limitations. First, it was a single-center retrospective study involving a relatively small sample size. Second, we did not assess pathological results. Strictly speaking, to evaluate pathological intratumoral fat accurately, we believe that “fat staining” is needed. We did not perform such a process, so we regard this as a limitation. Third, we did not evaluate PDFF maps or MR spectroscopy findings. Werven et al demonstrated that dual-echo MR imaging and MR spectroscopic measurements of hepatic fat had very strong correlations with histopathologic steatosis assessment outcomes in 46 patients’ livers (*r* = 0.85 and 0.86).^[15]^ Dual-echo CSI is proven to be equivalent to MR spectroscopy to measure liver fat; thus, we evaluated dual-echo CSI as a reference standard. Neither PDFF maps nor MR spectroscopy not required during our research period; however, we hope to compare FVF as measured by the MMD algorithm with these techniques in future work.

In conclusion, as compared with liver parenchyma, the correlations between FVF measured by a DECT-based MMD algorithm and FF as measured by CSI were weak in HCC in all phases. Although FVF is reproducible in HCC and liver parenchyma across scan phases by one-way ANOVA, the difference between non-CE FVF and four-CE FVF is higher in HCC than in liver parenchyma by Bland−Altman analysis. Overall, the MMD algorithm needs to be modified for “HCC fat quantification,” considering the heterogeneous various components of HCC.

## Author contributions

**Conceptualization:** Takashi Ota.

**Data curation:** Takashi Ota, Kosuke Sasaki.

**Formal analysis:** Takashi Ota.

**Funding acquisition:** Takashi Ota.

**Methodology:** Takashi Ota.

**Software:** Kosuke Sasaki.

**Writing – original draft:** Takashi Ota.

**Writing – review & editing:** Masatoshi Hori, Hiromitsu Onishi, Atsushi Nakamoto, Mitsuaki Tatsumi, Hideyuki Fukui, Kazuya Ogawa, Noriyuki Tomiyama.
